# Editorial: Cytoskeletal Regulation of Immune Response

**DOI:** 10.3389/fcell.2021.791327

**Published:** 2021-11-04

**Authors:** Sudha Kumari, Wolfgang W. Schamel, Balbino Alarcon

**Affiliations:** ^1^ The Department of Microbiology and Cell Biology, Indian Institute of Science, Bengaluru, India; ^2^ Department of Immunology, Faculty of Biology, University of Freiburg, Freiburg, Freiburg; ^3^ Centre for Chronic Immunodeficiency (CCI), University of Freiburg, Freiburg, Germany; ^4^ Signaling Research Centers BIOSS and CIBSS, Madrid, Spain; ^5^ Centro de Biología Molecular Severo Ochoa, Consejo Superior de Investigaciones Científicas, Universidad Autónoma de Madrid, Madrid, Spain

**Keywords:** immune cell cytoskeleton, actin dynamics, microtubules, immune response, immunological synapse

Perhaps the most characteristic property of both the adaptive and innate arms of our immune system is the ability to patrol basically the entire organism in search for potentially dangerous intruders, and respond once they are found. This response requires the activation of a complex machinery of intracellular pathways, the release of chemicals and proteins that act as chemoattractants to activate the migration of other cells of the immune system and finally, the coordination of a multicellular response that will lead to the destruction of the intruder. Immune cell mobility, immune cell activation and destruction of the intruder requires a precise spatiotemporal dynamics of the cells’ cytoskeleton. For this, the cell’s cytoskeleton must rearrange in a highly regulated fashion. Thus it is not surprising that these cells have developed an intricate network of cytoskeletal regulatory proteins which sometimes are exclusively expressed by cells of the immune system.

The cytoskeleton plays a versatile role in immune cells. A testament to this multifunctionality of cytoskeleton is that immune cells harboring mutations in cytoskeletal regulatory proteins often have compromised overall immune response. These defects exist even when the cells are challenged with potent activation signals *ex vivo*, suggesting that the function of the cytoskeleton is much more than just supporting migration to reach the intruder antigen, since a number of crucial immunoreceptor signaling functions are dependent on cytoskeletal organization and dynamics. However, the precise mechanistic roles that cytoskeleton plays in immune cell signaling, especially given that it is the same unit network that would perform all of the functions, remains a mystery.

In this special issue on the cytoskeleton in immune responses we have attempted to explore the reciprocal relationship between cytoskeleton, cellular signaling, and activation. In this issue there are original, opinion, and review articles that deepen our knowledge on the molecular regulation of the cytoskeleton as well as of cellular responses regulated by the cytoskeleton, with a goal to gain insights into the cytoskeleton—intracellular signaling machinery interconnection. These articles address this topic at diverse locations (membrane, nucleus, cytosolic organelles) and scales (molecular, organelle, cellular).

Immunopathies resulting from defects in cytoskeletal regulating proteins such as ArpC, HEM, WASP, WIP, and WDR1 serve as natural perturbations to study the regulation of the cytoskeleton. The review article by Dupré et al. describes how primary immunodeficiencies lead to distinct defects in immune cell function pointing to functional specializations of the cytoskeleton. Role of the actin microfilament NPF Wasp in antigen receptor signaling has been further elaborated in a review by Ngoenkam et al.. An article by Li et al. describes the role of WASP in regulating B cell actin architecture by forming actin-rich “pods” and cell spreading cells. Defects in WDR1, a regulator of cofilin activity, results in immunodeficiency. A report by Bolger-Munro et al. highlights the role of WDR-1 in actin remodeling and B cell responses.

This special issue covers cytoskeleton regulation and signaling in different lymphocyte types. The review by Ben-Shmuel et al. discusses NK cell cytoskeleton regulation and function, and dissects the role of individual cytoskeletal elements actin, microtubule, and NM-II in it. T cells require adhesion molecules such as integrin for activation, although their precise roles in signaling are not well understood. In a paper by Cassioli et al., the role of the integrin LFA1 at the T cell immunological synapse is explored using expression rescue experiments. The T cell line Jurkat E6.1 cells, which lack LFA1, showed restoration of architecture, signaling and activation when LFA1 was expressed at an adequate level.

Mechanical regulation of immune cell activation has gained popularity, since recent studies support the idea that the mechanical context of molecular interactions can influence the signaling output. In a report by Escolano et al., the authors find that altering the mechanical context of macrophage activation has profound effects on inflammasome signaling. Being a “novel” field the readouts (nature and magnitude of individual forces exerted at the molecular and cellular level) are still limited. A report from Wohl and Sherman uses spectral analysis to quantify ATP-dependent mechanical vibrations of plasma membranes during T cell activation. Further quantitative tools to analyze mechanics in immune cells are discussed by Fritzsche in an opinion article and by Garlick et al. focusing on cutting edge super resolution techniques and on interdisciplinary approaches to extract maximum spatiotemporal signaling information from cells. Along the same theme of using the experimental system to better extract information from cells, a paper by Lee et al. describes a method for the analysis of cell migration as a marker for T cell proliferation potential. In their review article, Record et al. discuss molecular mediators of mechanical force transduction in immune cells and emphasize a need for understanding actin dynamics in not just the cytoplasm of the cell as has been conventionally done, but also in the nucleus of the cell.

Regulation of the cytoskeleton in immune cells also involves uncharacterized and sometimes unconventional players. The precise mechanistic roles of these effectors are not completely clear. In the issue we publish articles that shed light on a few of these crucial players as regulators of subcellular localization and dynamics of filamentous actin. A review by Martín-Cófreces et al. discusses the role of the CCT chaperonin in the remodeling and positioning of the centrosome, and consequently in the dynamics of the immunological synapse. Similarly, a paper by Ibañez-Vega et al. analyses the role of the proteasome adaptor protein ECM 29 in proteosome localization and actin remodeling at the B cell immunological synapse. Another intriguing molecular player characterized here is Glial Matural Factor gamma (GMFgamma). A study by Deretic et al. uncovers a role of GMFgamma in cell spreading and actin remodeling and regtrograde flow at immunological synapse. Finally, a review by Kim et al. consolidates the knowledge about the role of transgelin in filamentous actin stabilization, and, consequently, in the stabilization of cellular microarchitectures such as microvilli.

Together, the issue covers a broad spectrum of articles on the cytoskeletal regulation in immune cells. In the future, we look forward to learn about additional players, novel mechanism on microscale architectures, emergent shape and its connection to cell functions.

